# Morpho-mechanics of human collagen superstructures revealed by all-optical correlative micro-spectroscopies

**DOI:** 10.1038/s42003-019-0357-y

**Published:** 2019-03-26

**Authors:** Raffaella Mercatelli, Sara Mattana, Laura Capozzoli, Fulvio Ratto, Francesca Rossi, Roberto Pini, Daniele Fioretto, Francesco Saverio Pavone, Silvia Caponi, Riccardo Cicchi

**Affiliations:** 1 National Institute of Optics, National Research Council (CNR-INO), Via Nello Carrara 1, I-50019 Sesto Fiorentino, Italy; 2 Department of Physics and Geology, University of Perugia, Via Alessandro Pascoli, I-06123 Perugia, Italy; 3 Institute of Applied Physics “Nello Carrara”, National Research Council (CNR-IFAC), Via Madonna del Piano 10, I-50019 Sesto Fiorentino, Italy; 4 Center of Electron Microscopy “Laura Bonzi” (Ce.M.E), Institute of Chemistry of Organometallic Compounds, National Research Council (CNR-ICCOM), Via Madonna del Piano 10, I-50019 Sesto Fiorentino, Italy; 5 CEMIN-Center of Excellence for Innovative Nanostructured Material, Via Alessandro Pascoli, I-06123 Perugia, Italy; 6 European Laboratory for Non-linear Spectroscopy (LENS), Via Nello Carrara 1, I-50019 Sesto Fiorentino, Italy; 7 Department of Physics, University of Florence, Via Giovanni Sansone 1, I-50019 Sesto Fiorentino, Italy; 8 Institute of Materials, National Research Council (CNR-IOM), Unit of Perugia, c/o Department of Physics and Geology, University of Perugia, Via A. Pascoli, I-06123 Perugia, Italy

## Abstract

In every biological tissue, morphological and topological properties strongly affect its mechanical features and behaviour, so that ultrastructure, composition and mechanical parameters are intimately connected. Overall, it is their correct interplay that guarantees the tissue functionality. The development of experimental methods able to correlate these properties would open new opportunities both in the biological and the biomedical fields. Here, we report a correlative study intended to map supramolecular morphology, biochemical composition and viscoelastic parameters of collagen by all-optical microscopies. In particular, using human corneal tissue as a benchmark, we correlate Second-Harmonic Generation maps with mechanical and biochemical imaging obtained by Brillouin and Raman micro-spectroscopy. The study highlights how subtle variations in supramolecular organization originate the peculiar mechanical behavior of different subtypes of corneal lamellae. The presented methodology paves the way to the non-invasive assessment of tissue morpho-mechanics in biological as well as synthetic materials.

## Abstract (web summary)

Raffaella Mercatelli et al. present a method incorporating Brillouin and Raman microscopy with Second-Harmonic-Generation microscopy. They examine human corneas, showing an unprecedented combination of viscoelastic, biochemical, and morphological properties of collagen.

## Introduction

The physiology and the functionality of biological tissues strongly depend on their architectural, biomechanical and biochemical features. The assessment of these properties is pivotal for providing an exhaustive characterization and for ascribing different functions to different biological structure^1,2^. It is also noticeable that in a broad variety of pathological conditions, dysfunctional alterations are often related to modifications in mechanics and biochemistry of cells and tissues^3^. A non-invasive contactless approach enabling their characterization at microscopic level holds clear strategic interest for both fundamental understanding and pathological assessment. The viscoelastic, biochemical and morphological properties of biological tissues can be respectively investigated by means of Brillouin^4–6^, Raman^7,8^ and Second-Harmonic Generation (SHG) microscopies^9–11^, as demonstrated by their large use for tissue characterization. Their integration, never reported so far, would provide an unprecedented combination of viscoelastic, biochemical and morphological information at microscopic length scales in label-free and contactless modality, opening a new frontier in bio-imaging.

Whereas conventional methods used for the analysis of the biomechanics of tissues generally require the application of mechanical forces and/or the use of contact probes, Brillouin micro-spectroscopy provides a powerful imaging alternative for the contactless and non-destructive determination of viscoelastic parameters. Based on the inelastic scattering of light from spontaneous acoustic waves present at thermal equilibrium in any material, this technique provides a direct measurement of the real and imaginary components of the high-frequency longitudinal elastic modulus^6,12,13^. Its contactless character, together with the high spatial resolution accessible also in depth, have made Brillouin microscopy a promising tool in the mechanical analysis of biological materials^14^. Brillouin spectroscopy has already proven to hold diagnostic power in ophthalmology^15^ and histopathology, based on its ability to distinguish different anatomical or subcellular structures by mechanical imaging^16–18^ and to reveal pathological conditions related to viscoelastic alterations^16,19–22^. Brillouin spectroscopy has already found application in the field of drug discovery, such as to convey information on the drug response of tumour cell aggregates^23^. The simultaneous collection, from the same scattering volume, of Brillouin and Raman signals over a broad frequency range, i.e., from fractions of GHz to hundreds of THz, is an innovative approach^24–26^ to correlate mechanical and biochemical properties in label-free condition with sub-micrometric spatial resolution. This combination has provided new knowledge insights into living cells, where mechanical modulations were associated to subcellular structures recognized by Raman markers^20,26^, as well as tissues, where chemical modifications induced by pathological conditions were shown to modify their biomechanics^27^.

The morpho-mechanical properties of biological tissues are mostly determined by collagen abundance and organization. As the fundamental building block of the extracellular matrix and of many biological tissues such as tendon, bones, cornea, blood vessels and cartilage, collagen is the most abundant structural protein in animals. Based on the hierarchical assembly of its triple alpha-helix units at the molecular scale (tropocollagen), at supra-molecular level, collagen is organized in various structures displaying specific morphological, mechanical and biological properties. It turns out that polarization-resolved SHG data hold the key for an accurate morphological characterization of collagenous tissues. In fact, SHG signals encode the supramolecular symmetry of collagen superstructures in a unique way, by probing their second-order non-linear optical response. In particular, SHG microscopy is ideal to highlight molecular changes as well as architectural remodelling of collagenous stroma^28^, which plays a key role in connective tissue diseases^29^, fibrosis^30^, as well as in tumour development from in-situ to invasive stage^31^.

One of the most intriguing collagen-rich tissues is the cornea. The complex corneal architecture, its hierarchical organization, its intricate arrangement of collagenous lamellae has attracted enduring interest from the scientific community aimed to explain the peculiar optical and mechanical properties of this tissue. In recent years, experimental efforts revealed new details of its structure, which is far more heterogeneous than previously thought^32^. For example, there exists, in the anterior portion of the stroma of healthy human corneas, a particular class of collagen structures, called sutural lamellae. Their name indicates their function, which is to link the outermost Bowman’s layer to deeper portions of corneal stroma, by embracing other collagen fibres. The key role played by these structures, running in multiple directions, often branching out and intertwining in irregular patterns^33^ while preserving corneal shape and curvature, was the focus of several previous studies. For example, 3-Dimensional SHG imaging showed that, in pathologies such as keratoconus where the curvature of corneal surface deforms into a conoid, the density of sutural lamellae is much lower than normal^34^.

Here, we propose a method based on a correlative combination of three imaging techniques on ex-vivo human corneal tissue, which is a representative benchmark to highlight the overwhelming relationship that exists in nature between structure, function, and mechanical properties. The synergic integration of Brillouin, Raman and SHG microscopy yields an unprecedentedly comprehensive insight into the morpho-mechanical/chemical features of corneal samples. In particular, we demonstrate that the heterogeneous pattern of collagen superstructures, featuring sutural and standard lamellae, corresponds to a diverse supramolecular symmetry, as seen in SHG data, and a diverse stiffness, as highlighted by Brillouin maps, but not to a chemical modulation, as revealed by Raman spectra. Thanks to a general model of the second order susceptibility tensor, applied for the first time—to our knowledge—to corneal tissue, we analysed the morphological structure in both sutural and standard lamellae, and gained a detailed interpretation of their ultrastructure, consistent with electron microscopy data. The proposed correlative method yields an all-optical non-invasive assessment of tissue morphology, biomechanics and biochemistry.

## Results

### Correlative Raman, Brillouin and SHG analysis

**Fig. 1:**
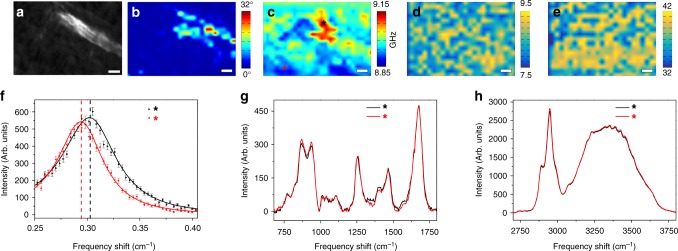
Correlative SHG, Brillouin and Raman imaging on collagenous stroma. **a** Maximum intensity projection of a SHG image stack acquired within collagenous corneal stroma using en-face optical sectioning geometry within a depth range of 10 μm starting from 10 μm below Bowman’s membrane. **b** Corresponding lamellar inclination map, obtained after a 3D correlation analysis^35^, depicted in a color-coded scale. **c** Brillouin map of the measured frequency shift, acquired in the same region as images in (**a**) and (**b**). **d** Corresponding Raman map of the OH stretching modes, represented as intensity ratio between the OH stretching region (3100–3800 cm^−1^) and the amide I region (1600–1750 cm^−1^). Mean error 6%. **e** Same as d) for the CH stretching band (2800–3100 cm^−1^). Mean error 6%. Scale bars: 10 μm. **f** Brillouin and (**g**) and (**h**) Raman spectra of two selected points indicated by black and red stars in the Brillouin map (**c**)

Figure 1 displays the results of our correlative analysis. In particular, Fig. 1a shows the maximum intensity projection of a SHG image stack acquired within bulk corneal tissue using coronal, i.e., en-face, optical sectioning, which highlights collagen morphology with high contrast and zero-background. The bright collagen lamella is identified as a sutural lamella because of its typical orientation at large angles with respect to corneal surface. The lamellar orientation map, shown in Fig. 1b was reconstructed by using epi-detection and applying a three-dimensional auto-correlation analysis^35^ within the image stack. The same tissue region was also probed by joint Brillouin and Raman micro-spectroscopy^20,24^. Site matching was enabled by referencing to a laser ablation pattern in the tissue epithelium, as described in section Methods. In order to make sure to investigate the intact corneal tissue and avoid any artefact that may originate from the ablation process, the correlative SHG, Raman and Brillouin investigation was performed several diameters away from every fiducial spot.

Representative spectra acquired in two different voxels are reported in Fig. (1f) showing that, while the fluctuations of Raman signals are homogenously distributed throughout corneal stroma, the frequency of Brillouin peaks (ω_b_) displays a prominent pattern. Since the longitudinal elastic modulus (M′) scales with the square of ω_b_^27^, the Brillouin image reported in Fig. (1c) implies a clear mechanical modulation.

In more detail, a quantitative assignment of M’ from ω_b_ requires knowledge of the parameter *ρ*/*n*^2^, i.e., M′ = (*λ*_i_/4*π*)^2^*ρ/*n*^2^ω_b_^2^ where *ρ* is density, n refractive index and *λ*_i_ the wavelength of the incident light (See section Methods). Even if refractive index and density are not spatially uniform throughout corneal stroma, their local variation tends to compensate in the ratio *ρ*/*n*^2^, which happens to vary by less than 0.3% in humans as well as in bovines^36^, i.e., less than an order of magnitude smaller than the span of Brillouin frequency variation observed in our maps. Therefore, we safely neglect this effect in our analysis and practically assume that M′∝ ω_b_^2^.

The heterogeneity in the stiffness shown in Fig. 1 displays a striking conformity with the SHG signal (Fig. 1a), thus highlighting, for the first time, to our knowledge, the ability of Brillouin spectroscopy to detect individual sutural lamellae as an elastic modulation inside the tissue. The simultaneous Raman maps show a slight inhomogeneity of the biochemical components (Figs. 1d, e), which does not correlate with the mechanical modulation. The Raman imaging of: (i) the OH signal (centred around 3500 cm^−1^) normalized to the amide 1 signal (centred around 1660 cm^−1^) and (ii) the CH stretching band (centred around 2950 cm^−1^) normalized to the same amide 1 signal, respectively associated to water vs protein and lipid vs protein content^37^, are insensitive to the presence of lamellae. This result suggests that the enhancement of the elastic modulus found in sutural lamellae is associated with morphological rather than biochemical factors.

### Viscoelastic properties revealed by Brillouin imaging

**Fig. 2:**
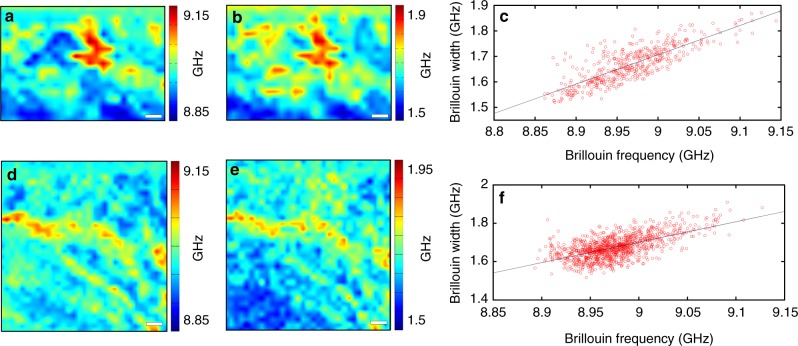
Correlative analysis on Brillouin peak frequency and width. **a** Maps of the Brillouin frequency shift and (**b**) of the relevant width acquired over the same position around a sutural lamella. The scales in the x and y axes are in µm, while the codes of colour are in GHz. **c** Linear relation (Pearson’s *r* = 0.81) between width and frequency relative to the maps in (**a**) and (**b**). **d**, **e** and **f** Same as (**a**), (**b**) and (**c**) for another field of view (Pearson’s *r* = 0.62, in this case)

Another feature that is already visible from the raw data reported in Fig. 1f is that the modification of the Brillouin peaks does not only involve their position but also their width, 2Γ_b_. A careful examination of the spectral shape reveals a correlation between ω_b_ and Γ_b_ that occurs in all Brillouin maps acquired over different locations of the cornea. As a rule of thumb, Γ_b_ clearly increases in stiffer parts of the tissue. As an example, two of the investigated regions are reported in Fig. 2, where the maps of ω_b_ (Figs. 2a, d) and Γ_b_ (Figs. 2b, e) are represented together with their mutual variation (Figs. 2c, f). While the real part of the longitudinal modulus, M′, proportional to the square of ω_b_, is an indicator of stiffness, its imaginary part, M′′, relates to Γ_b_ and accounts for the viscous properties, according to M′′ = ω_b_η_L_ = ω_b_Γ_b_*ρ*/*q*^2^, *q* = 2*n* k_i_ being the exchanged momentum, *n* the refractive index of the sample and **k**_i_ the wavevector of the incident light. The correlative analysis of Γ_b_ and ω_b_ provides insight into the viscoelastic nature of the material^21^. In particular, a positive correlation between these parameters has already been observed in highly hydrated materials like living or fixed cells^20,22^, while the opposite behaviour was found on dry samples such as epithelial tissue of Barrett’s oesophagus^19^ and amyloid plaques of transgenic mouse brain^21^. These results highlight the role of water, which is able to modify the viscoelastic response of materials by decreasing the main molecular relaxation times through a plasticizing effect^19^.

In the corneal tissue we find the increase of both stiffness and viscosity associated to the presence of sutural lamellae. An increase by 30% in Γ_b_ as evidenced in Figs. 2c, f corresponds to an increase by 30% in the molecular relaxation times within sutural lamellae, confirming their more rigid and arrested structure.

### In-depth correlative imaging and analysis

**Fig. 3:**
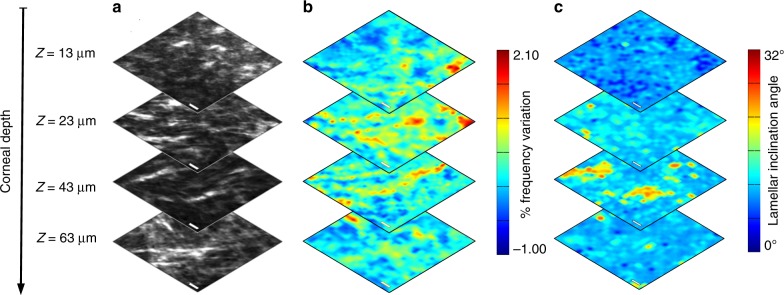
Correlative SHG-Brillouin axial optical sectioning analysis on corneal stroma. Comparison between (**a**) SHG images, (**b**) relative variations of Brillouin frequency and (**c**) lamellar inclination maps, acquired at the specified depths below Bowman’s membrane. Scale bars: 10 μm

**Fig. 4:**
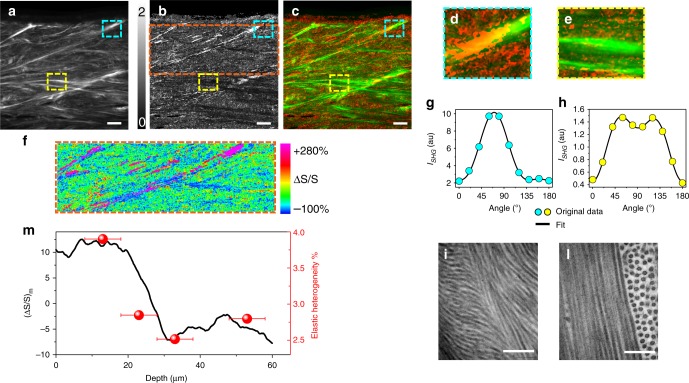
Morpho-mechanical properties of collagen lamellae with different supramolecular organization. **a** SHG image acquired on a sagittal section of the anterior corneal stroma in epi-detection optical configuration at a depth of 10 µm from the surface. Scale bars: 20 µm. **b** Symmetry map of the same field of view. **c** Merger of the SHG image (coded in green) with the symmetry map (red). Two details of the image, taken in the upper and in the lower stroma as indicated by the two insets framed in cyan and yellow, are presented with higher magnification in (**d**) and (**e**), respectively. **f** Inset corresponding to the orange rectangle in (**b**) showing the 80 μm-thick layer below Bowman’s membrane in a color-coded scale representing the deviation of the symmetry parameter from its average. **g** P-SHG profiles typical of trigonal (**g**, cyan dots) or cylindrical (**h**, yellow dots) symmetry, obtained from the lamellae represented in (**d**) and (**e**), with the corresponding fit functions superimposed as black lines. S = 1.6 and R = 0.01, where R is residue in (**g**). S = 0.01 and R = 0.001 in (**h**). **i** and **l** TEM images taken in the upper (**i**) and deeper (**l**) stroma showing the supramolecular organization of collagen fibrils in sutural and flat lamellae, respectively. Scale bars: 200 nm. **m** Relative variation of the symmetry parameter versus stromal depth, represented as percent variation with respect to the average calculated from the symmetry map in (**f**). This variation is compared with the elastic heterogeneity measured by Brillouin scattering at four different depths. The error bars in the elastic heterogeneity correspond to the axial size of the scattering volume

Additional insight originates from a comparison of Brillouin and SHG optical sectioning. We found that the correlation between the presence of sutural lamellae and the elastic modulation persists throughout the probed tissue (Figs. 3a, b), but the elastic heterogeneity, calculated as the ratio of standard deviation to average of ω_b_ at any given depth, decreases from about 4 to 2.5% when imaging deeper into corneal stroma. However, this result does not correlate well with lamellar inclination alone (Fig. 3c) and so cannot be ascribed to the elastic anisotropy of collagen fibres^12^, but rather supports the hypothesis of an ultrastructural singularity of sutural lamellae. To gain insight into this issue, we analysed the supra-molecular organization of collagen using P-SHG microscopy^28,38,39^, as described in detail in the section Methods. Figure 4a shows an SHG image of a thin sagittal optical section of the upper part of corneal stroma. The SHG polarization map of the same region is reported in Fig. 4b in terms of the symmetry parameter S = |χ_yyy_|/χ_zxx_, which is an indicator of lattice system vanishing in the cylindrical limit of ideal parallel fibres (see section Methods).

Figure 4c was obtained by their merger and denoting S in red and the SHG intensity in green. Interestingly, we found that higher values of S match with sutural lamellae, whereas quasi-null values pertain to flat lamellae. Even when their SHG intensity is very similar, S remains higher in sutural (Fig. 4d) as compared to flat lamellae (Fig. 4e). By analysing the measured SHG polarization profile in detail, we found shapes ranging from a symmetric profile (Fig. 4h) corresponding to values of S around zero to more and more asymmetric shapes characterized by increasing values of S (Fig. 4g). This diverse optical behaviour points to a different symmetry of the local ultrastructure at a supramolecular scale: the symmetric P-SHG profile of flat lamellae is consistent with cylindrical symmetry and the asymmetric P-SHG profile of sutural lamellae implies its break into trigonal symmetry.

A confirmation of this result comes from electron microscopy. Flat collagen lamellae located deep-inside corneal stroma (Fig. 4l) consist of adjacent collagen fibrils oriented in the same direction and staggered side-by-side, thus satisfying the requirements for cylindrical symmetry. Conversely, a TEM micrograph of sutural lamellae found in the anterior portion of corneal stroma (Fig. 4i) shows a helicoidal distribution of collagen fibrils, in agreement with trigonal symmetry. Finally, by analysing the mean variation of S with depth (Fig. 4m), corresponding to the symmetry map in Fig. 4f), we found a transition from a positive to a negative shift from the average. This behaviour, typical of all the examined corneal samples (see Supplementary Figure 2), clearly correlates with the elastic heterogeneity measured by Brillouin scattering and reported in the same Figure. As a consequence, the modification of mean lamellar ultrastructure found from Bowman’s membrane to deeper corneal stroma is interpreted as the microscopic origin of the measured elastic modulation.

## Discussion

The present study combines SHG, Brillouin and Raman microscopies to noninvasively unravel the complex relationship between morphological, mechanical and chemical composition from within biological tissues, with microscopic optical resolution. The successful test of our method is provided in the significant case of human corneal collagen. Brillouin studies on different corneal samples were already reported in the literature^40^ and disclosed a decreasing trend of rigidity with stromal depth^36,41,42^ or evidenced mechanical modifications induced by the pathological conditions^15^. To the best of our knowledge, here, we have found, for the first time, a much richer heterogeneous scenario affecting all spatial coordinates in the healthy cornea. The analysis of Brillouin maps in a non-fixed human cornea (see Supplementary Figure 1) shows the same mechanical modulation, thus confirming that its origin is the intrinsic tissue morphology, independent of sample preparation.

For a coronal section at a given depth inside a sample, we have identified a correlation between a modulation of viscoelastic properties, both in terms of frequency and width of the Brillouin peak, and the presence of different collagen structures in sutural lamellae. This achievement was enabled by the unique features of our custom-built Brillouin microscope^20,24^, which, at the expense of longer acquisition time, combines highest resolution and contrast. Moreover, the use of a correlative approach was key to achieve a deeper morphological interpretation of the mechanical properties, otherwise inaccessible to ordinary methods based on a single mechanism of optical contrast. In particular, as the Brillouin maps revealed a heterogeneity in stiffness and viscosity at the length scale of individual lamellae, it was its correlative analysis with SHG images that unambiguously linked this viscoelastic modulation to the presence of sutural lamellae, while Raman microscopy simultaneously excluded any biochemical implication.

The in-depth correlative analysis demonstrates that the peculiar mechanical properties of sutural lamellae are not related to their particular orientation. It is well known that the elastic tensor of collagen fibres features different elastic constants, so that, by probing different directions, the frequency position of Brillouin peaks undergoes a modulation and reaches its maximum along the fibre longitudinal axis^12,43,44^. However, in the present case, as shown by data reported in Fig. 3, the elastic heterogeneity reaches its maximum in the very proximity of Bowman’s layer, where sutural lamellae take a direction parallel to corneal surface. In this condition, by en-face optical sectioning in back-scattering geometry, Brillouin spectroscopy probes the fibre transversal axis. Therefore, there is little correlation between the overall fibre orientation and the observed elastic modulation. Instead, we ascribe the latter to the internal conformation of collagen lamellae.

The general model of the second order susceptibility tensor here applied for the first time, to our knowledge, to the analysis of corneal tissue revealed a distinctive transition of collagen assembly in sutural lamellae, which highlights the relationship between ultrastructural heterogeneity and elastic properties in connective tissue.

By using non-contact non-invasive optical techniques, our method is easily applicable to ex-vivo biological tissues, to  clinical diagnostic purposes, as well as to the analysis of broad classes of biological and synthetic materials. Widespread interest is emerging around the application of light and optical techniques in the analysis of biological materials. Their ability to characterize systems over multiple length scales ranging from subcellular compartments to whole tissues puts optical techniques in an ideal position to pursue insight in fundamental biology as well as to develop tools for the clinical practice.

Regarding a potential future translation of the proposed technology into the clinical practice, some considerations are relevant. Brillouin imaging has already been implemented for diagnostic purposes in patients^15^ by using an exposure time and laser power density safe for corneal tissue. Our method adds the detection of Raman signals, thus gaining sensitivity to biochemical composition, without any additional radiation dose. Also, polarization-resolved SHG imaging of corneal stroma has already been applied in-vivo in animal models^45^ and, more recently, also in human subjects^46^, with a laser power density complying with ANSI limits.

The correlative combination of these methods may pave the way to new clinical applications, such as an early detection of a variety of pathological conditions as well as for treatment follow-up. In particular, a precise characterization of collagen fibrils in the anterior part of corneal stroma is of great interest to understand the aetiology of rare conditions, such as keratoconus, or other dystrophies related to subtle morphological deformations. As an example, an early diagnosis of keratoconus may prevent the need for corneal transplantation and a contactless observation of collagen morpho-mechanics may help to follow treatments based on corneal collagen cross-linking (CXL)^47,48^ or in tandem with laser in-situ keratomileusis (LASIK) as a tool to re-establish corneal biomechanics^49–51^.

More in general, we propose a new investigation method that is able to correlate morphological, chemical and mechanical properties in biological tissues opening a new frontier in bio-imaging with a strong potential for impact in all clinical procedures requiring a non-invasive assessment of collagen morphology and biomechanics.

## Methods

### Sample preparation

This study was conducted in accordance with the tenets of the Declaration of Helsinki. Healthy human corneas were purchased from *Veneto Eye Bank* Foundation (Venice, Italy), upon selection among those not suitable for transplantation. After collection, samples were maintained in a tissue preservation solution (CARRY-C, Alchimia srl, Padova, Italy) for a maximum of three days from delivery and then treated according to a protocol that ensures the preservation of its mechanical integrity and morphology^52^. First, samples were fixed for 24 h in phosphate-buffered saline with 4% paraformaldehyde and rinsed with the same buffer before preparation for imaging. Corneal button were then separated from the sclera by a Barron Donor Cornea Punche (Katena Products, Inc. Court Denville, New Jersey, USA) and inserted into a home-made chamber made by sandwiching two glass coverslips sealed with glue and filled with phosphate-buffered saline, in order to maintain physiological osmolarity. One sample, presented in Supplementary Figure 1, was analysed before any fixation protocol.

**Fig. 5:**
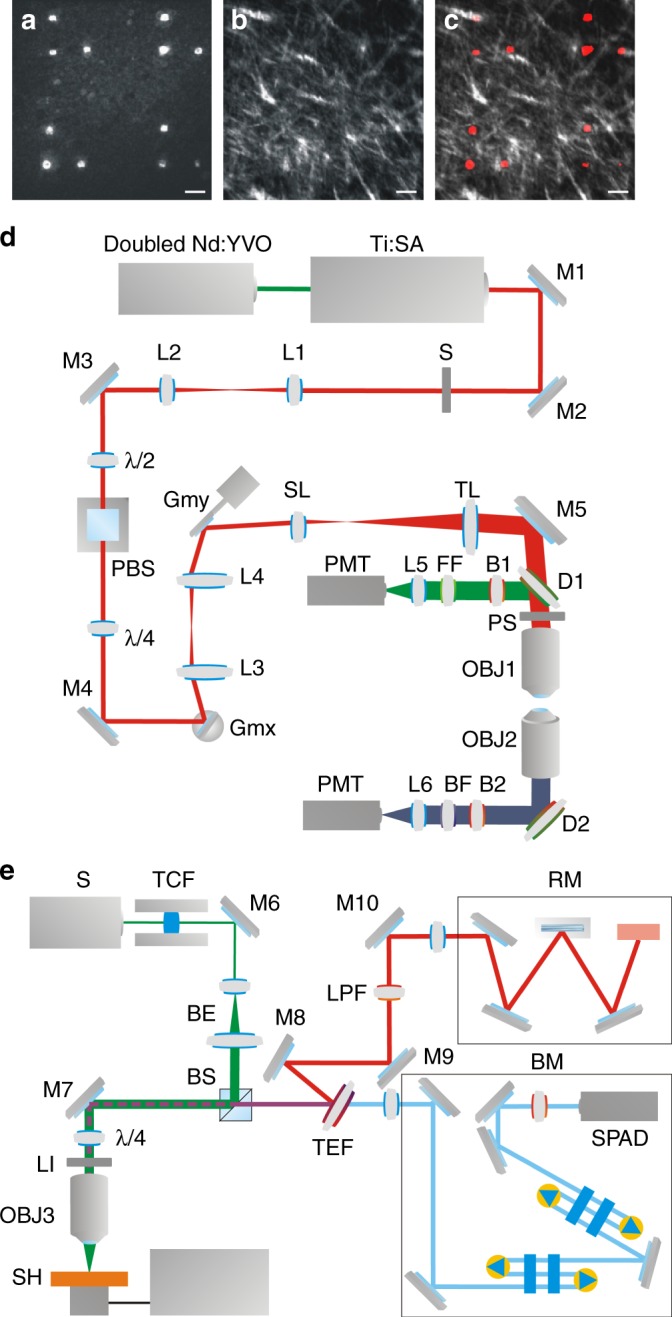
Experimental setups for correlative SHG, Brillouin and Raman microscopy. **a** Corneal epithelium imaged by two-photon fluorescence displaying a laser ablation pattern used as fiducial markers. Scale bars: 60 μm. **b** Maximum intensity projection of a SHG image stack acquired in the corneal stroma below the laser ablation markers, showing the intact pattern of collagen lamellae. **c** Merge of the images shown in (**a**) and (**b**) with fluorescence coded in red and SHG in gray. **d** Optical scheme of the experimental setup used for SHG microscopy. The laser excitation path is depicted in red, while the detection paths are depicted in green and blue for fluorescence and SHG, respectively. (*L* lens, *M* mirror, *S* shutter, *λ*/2 half wave plate, *PBS* polarizer, *λ*/4 quarter wave plate, *Gm* galvanometric mirror, *SL* scanning lens, *TL* tube lens, *D* dichroic mirror, *PS* polarization scanner, *OBJ* objective lens, *B* laser blocking filter, *FF* fluorescence filter, *BF* band-pass filter, *PMT* photomultiplier tube). **e** Optical scheme of the experimental setup used for Brillouin and Raman microscopy (BM and RM), consisting of a confocal microscope, a high contrast tandem Fabry-Perot interferometer and a Raman spectrometer. *LS* laser source, *TCF* temperature-controlled etalon, *M* mirror, *BE* beam expander, *BS* broadband polarizing beam splitter, *λ*/4 quarter wave plate, *LI* coaxial LED illuminator, *OBJ* objective lens, *SH* three-axis piezoelectric translation stage, *TEF* tunable ultrasteep short-pass filter, *TFP-2-HC* Brillouin spectrometer Tandem Fabry Perot-2-High Contrast, *SPAD* single-photon avalanche photodiode, *LPF* long-pass filter, *RM* Raman spectrometer)

In order to precisely image the same field of view with SHG, Brillouin, and Raman microscopy, a distinctive pattern of marks was burned in the corneal epithelium by using laser ablation, as shown in Fig. 5a). Each mark was produced as a photothermal damage by using a laser power of about 30 mW and by illuminating an area of 10 × 10 μm^2^. The spatial confinement of the ablation and the absence of damage on the underlying collagenous stroma was verified by acquiring an image stack in the uppermost stroma, immediately below Bowman’s membrane, by SHG microscopy (Fig. 5b). Figure 5c shows a merger of the images in Figs. 5a, b. In order to investigate intact corneal tissue and avoid any possible influence of the ablation process, the correlative SHG, Raman and Brillouin investigation was performed at least 60 μm along a radial direction and several tens of microns along the optical axis away from every fiducial spot.

### SHG imaging setup

A custom-made laser-scanning non-linear microscope, mounted on a vertically placed stainless steel honeycomb breadboard, was used for SHG imaging (Fig. 5d). The laser source was a mode-locked Ti:Sapphire laser (Mira 900 F, Coherent Inc., Santa Clara, CA, US) pumped by a frequency-doubled Nd:YVO_4_ laser system, emitting at 532 nm (Verdi V10, Coherent Inc., Santa Clara, CA, US). For imaging collagen with SHG, the excitation wavelength was tuned to 840 nm. After a collimating telescope (L1, L2) and an electronic shutter (S), a system consisting of a rotating half-wave plate (*λ*/2) and a polarizer (PBS) was implemented to finely adjust the laser power, whereas a quarter-wave plate (*λ*/4) was used to control the polarization state in the back focal plane of the objective lens and to compensate for aberrations introduced by optical components. Either linear or circular polarization were used in this study, in order to respectively obtain a SHG response that is polarization-independent or polarization-dependent.

The laser beam was raster scanned on the sample by means of two orthogonal galvanometric mirrors (Gmx, Gmy, VM500, GSI Lumonics, Karlsruhe, Germany), optically relayed by two lenses (L3, L4, *f* = 60 mm) in a 4 f configuration. After passing through the scanning head, the laser beam was expanded to a size around 1 cm by means of a telescope, made by the scanning (SL) and the tube lens (TL), before focusing by a Plan-Apochromat ×40 objective lens (OBJ1, NA 1.4, WD 0.13 mm, Carl Zeiss Microscopy, Jena, Germany). For polarization-sensitive acquisition, a polarization scanner, realized with a half-wave plate inserted in a motorized rotating mount, was placed immediately before the objective diaphragm, in order to excite the sample with linear polarization of controllable orientation. The fluorescence emitted by the sample was collected by the same objective lens used for the excitation, reflected by a dichroic mirror (D1, 685DCXRU Chroma Technology Corporation, Rockingham, VT, US) and directed into a photomultiplier tube (PMT1, H7422 Hamamatsu, Hamamatsu City, Japan). Forward-emitted SHG was collected by a water immersion objective lens Fluor ×40 (OBJ2, NA 0.8, WD 2 mm, Nikon Corporation, Tokyo, Japan), reflected by a dichroic mirror (D2, 685DCXRU Chroma Technology Corporation, Rockingham, VT, USA) and detected by a photomultiplier tube (PMT2, H7422 Hamamatsu, Hamamatsu City, Japan). A narrow band-pass filter centred at 420 ± 10 nm (BF, HQ420BP Chroma Technology Corporation, Rockingham, VT, US) was placed in the detection path in order to selectively detect second-harmonic light. Two short-pass filters were inserted in the fluorescence and SHG detection paths (B1,B2, E700SP-2P Chroma Technology Corporation, Rockingham, VT, US) in order to reject any scattered laser light.

### SHG image acquisition and analysis

SHG images and stacks were collected from the central portion of corneal samples, using en-face optical sectioning geometry (optical axis orthogonal to tissue surface) and forward detection scheme. Imaging parameters were: a field of view of 200 × 200 μm^2^, a resolution of 1024 × 1024 pixel^2^, a pixel dwell time of 20 μs, a wavelength of 840 nm, and an average laser power of about 8 mW. The spatial resolution was about 300 nm in the radial direction and 1000 nm along the optical axis. In order to correlate the SHG images with those from the Brillouin—Raman microscope, we applied a spatial binning on the SHG data to realize an effective resolution matching that obtainable by the other two techniques. The mean inclination of sutural lamellae that are visible in SHG stacks was quantified by means of an autocorrelation-based method^35^.

**Fig. 6:**
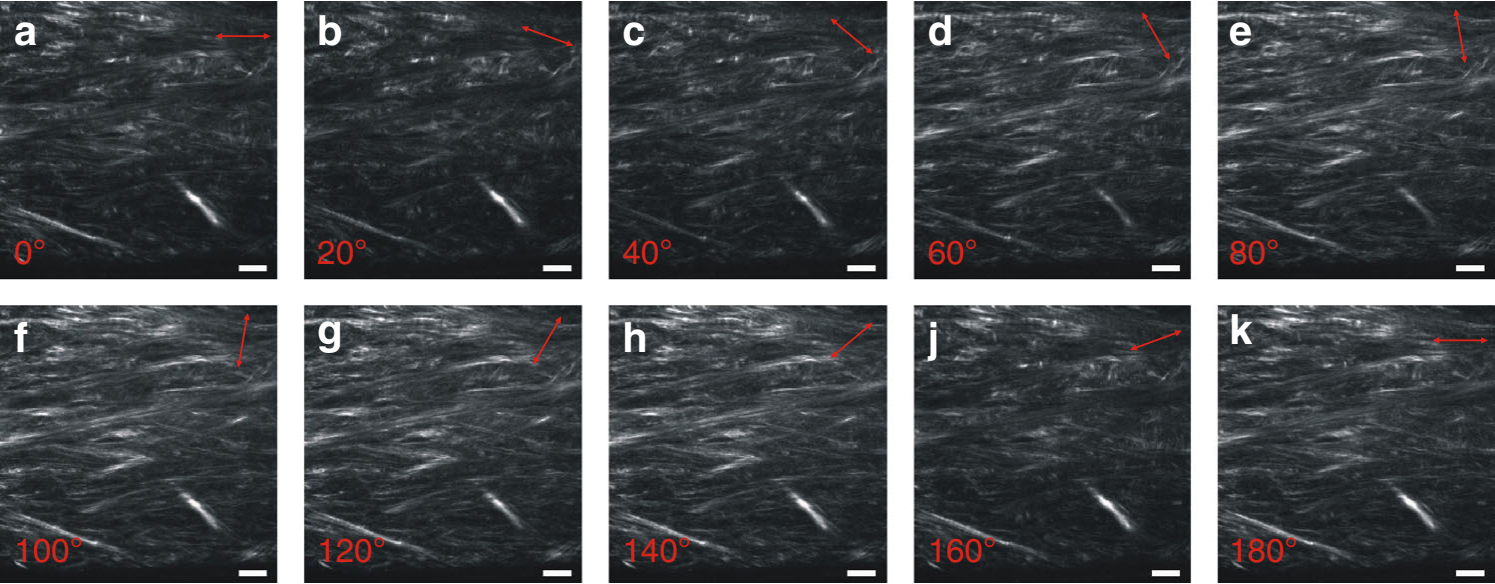
P-SHG Image stack. Example of a P-SHG image stack taken from a sample of human cornea imaged using sagittal optical sectioning geometry. The polarization direction is rotated in steps of 20°, as schematically displayed by the red arrows insets in the top right corner of the images. The orientation of the polarization with respect to the image horizontal axis is as follows: **a** 0°, **b** 20°, **c** 40°, **d** 60°, **e** 80°, **f** 100°, **g** 120°, **h** 140°, **i** 160°, **j** 180°. Field of view: 200 × 200 μm^2^. Resolution: 1024 × 1024 pxl^2^

The laser polarization in the BFP was circular in most cases and linear only for the P-SHG measurements, where its orientation was varied in steps of 20° by rotating a half-wave plate (see Fig. 6 for a typical acquisition). The resulting image stacks were analysed by a custom analysis routine written in LabVIEW (National Instrument, Austin, TX, US), in order to fit the polarization-intensity curves from each pixel with the following function:

$${\mathrm{I}}^{\mathrm{SHG}} = {\mathrm{A}}\left( \left( {\mathbf{d}}_{\mathbf{22}} {\mathrm{sin}}^{2} \alpha + {\mathbf{d}}_{\mathbf{15}} {\mathrm{sin}} \hskip3pt 2 \alpha \right)^{2} + \left( {\mathbf{d}}_{\mathbf{31}} {\mathrm{sin}}^{2} \alpha + {\mathbf{d}}_{\mathbf{33}} {\mathrm{cos}}^{2} \alpha \right)^{2} \right),$$

where d_**22**_ = χ_yyy_, d_**15**_ = χ_xxz_, d_**31**_ = χ_zxx_, d_**33**_ = χ_zzz_, are elements of the second order susceptibility tensor, α is the angle between fibrillar axis and polarization orientation, and A is a normalization factor. Since χ_yyy_ departs from zero and increases in absolute value as trigonal symmetry emerges over cylindrical symmetry (see the following paragraph), we defined a symmetry parameter that captures this trend. In order to increase the sensitivity of this parameter with respect to collagen supramolecular symmetry, we used the ratio between the module of χ_yyy_ and χ_zxx_, i.e., S = |d_**22**_|/d_**31** **=** _|χ_yyy_ |/χ_zxx._ We reconstructed spatially resolved maps of S by analysing each pixel.

### P-SHG mathematical model

In non-linear optics, second-order optical processes, such as SHG depend on the second order polarization, which is given by the equation:

1 $$P_i\left( {2\omega } \right) = 2\mathop {\sum }\limits_{jk} \mathop {\sum }\limits_{nm} \chi _{ijk}\left( {\omega _n + \omega _m,\omega _n,\omega _m} \right)E_j(\omega _n)E_k(\omega _m),$$

Where ω_n_ and ω_m_ are two distinct frequencies of the incident field, *χ*_*ijk*_ are elements of the second order susceptibility tensor **χ** (*i,j,k*) corresponding to the directions of the principal axes, *E*_*j*_(*ω*_*n*_) and *E*_*k*_(*ω*_*n*_) are the amplitudes of the electric field components at the frequency ω_n_ and ω_m_, respectively.

For SHG, ω_n_ = ω_m_ = ω and ω_n_ + ω_m_ = 2ω, so that the equation for the polarization reads:

2 $$P_i\left( {2\omega } \right) = 2\mathop {\sum }\limits_{jk} \chi _{ijk}\left( {2\omega ;\,\omega ,\omega } \right)E_j(\omega )E_k.$$

*χ* is a rank 3 matrix of 27 elements that can be simplified to a matrix of 18 non-vanishing elements due to symmetry in the last two indices (since ω_n_ = ω_m_ = ω in SHG)^53^

3 $$\left( {\begin{array}{*{20}{c}} {P_x} \\ {P_y} \\ {P_z} \end{array}} \right) = 2\left( {\begin{array}{*{20}{c}} {\chi _{xxx}} & {\chi _{xyy}} & {\chi _{xzz}} & {\chi _{xyz}} & {\chi _{xxz}} & {\chi _{xxy}} \\ {\chi _{yxx}} & {\chi _{yyy}} & {\chi _{yzz}} & {\chi _{yyz}} & {\chi _{yxz}} & {\chi _{yxy}} \\ {\chi _{zxx}} & {\chi _{zyy}} & {\chi _{zzz}} & {\chi _{zyz}} & {\chi _{zxz}} & {\chi _{zxy}} \end{array}} \right)\left( {\begin{array}{*{20}{c}} {E_xE_x} \\ {E_yE_y} \\ {E_zE_z} \\ {2E_yE_z} \\ {2E_xE_z} \\ {2E_xE_y} \end{array}} \right).$$

In previous studies^54,55^, focused on the characterization of collagen fibrils in corneal samples, P-SHG data were modelled with a simplified formulation of **χ** derived from the assumption of Kleinman symmetry for collagen fibrils. This condition implies the free commutability of all three indices *i,j,k*. Moreover, the assumption of cylindrical symmetry in the spatial arrangement of the harmonophores reduces the number of non-vanishing terms^56^. Under these assumptions, **χ** takes the form:

4 $$\left( {\begin{array}{*{20}{c}} 0 & 0 & 0 & 0 & {\chi _{xxz}} & 0 \\ 0 & 0 & 0 & {\chi _{xxz}} & 0 & 0 \\ {\chi _{xxz}} & {\chi _{xxz}} & {\chi _{zzz}} & 0 & 0 & 0 \end{array}} \right).$$

Although Kleinman symmetry is frequently assumed for modelling the P-SHG response of collagen, it has been demonstrated that its validity fails in several applications^57^. The reason for this failure is that, in most practical contexts, Kleinman symmetry is only partially fulfilled. This happens when the electronic resonance frequencies of the harmonophores are less than about an order of magnitude greater than the driving frequency. The second assumption is also most frequently used in P-SHG experiments and relies on the symmetry in the spatial distribution of the harmonophores, which is supposed to be cylindrical. The condition of cylindrical symmetry is satisfied when collagen fibrils are staggered side-by-side and parallel to each other^58^. Conversely, when this ideal fibrillar geometry is not respected, the assumption of cylindrical symmetry immediately fails. If collagen fibrils assemble in a more general helicoidal distribution, a trigonal symmetry (3 m) is a more appropriate model to describe their SHG. Under the hypothesis of trigonal symmetry, *χ* takes the new form:

5 $$\left( {\begin{array}{*{20}{c}} 0 & 0 & 0 & 0 & {\chi _{xxz}} & { - \chi _{yyy}} \\ { - \chi _{yyy}} & {\chi _{yyy}} & 0 & {\chi _{xxz}} & 0 & 0 \\ {\chi _{zxx}} & {\chi _{zxx}} & {\chi _{zzz}} & 0 & 0 & 0 \end{array}} \right).$$

The expression [5] differs from [4] by element *χ*_*yyy*_, which is non-vanishing, and by the inequality: *χ*_*xxz*_ ≠ *χ*_*zxx*_. Element *χ*_*yyy*_ takes a non-vanishing value that increases in absolute terms as the trigonal symmetry prevails over cylindrical symmetry. The failure of Kleinmann symmetry instead implies that *χ*_*xxz*_ ≠ *χ*_*zxx*_.

By assuming a driving field propagating along *x*, a collagen fibril oriented along *z* and an electric field polarized along a direction that forms an angle α with respect to the fibrillar axis, the electric field vector reads:

6 $$E_z\left( \omega \right) = E\left( \omega \right)\cos \left( \alpha \right).$$

7 $$E_y\left( \omega \right) = E\left( \omega \right)\sin \left( \alpha \right).$$

8 $$E_x\left( \omega \right) \cong 0.$$

In this approximation, the components of the polarization vector read:

9 $$P_x\left( {2\omega } \right) = 2\chi _{xxz}E_x\left( \omega \right)E_z\left( \omega \right) - 2\chi _{yyy}E_x\left( \omega \right)E_y\left( \omega \right) \cong 0.$$

10 $${P_y\left( {2\omega } \right) = \chi _{yyy}\left[ {\left( {E_x\left( \omega \right)} \right)^2 - \left( {E_y\left( \omega \right)} \right)^2} \right] + 2\chi _{xxz}E_y\left( \omega \right)E_z\left( \omega \right) \cong 2\chi _{xxz}E_y\left( \omega \right)E_z\left( \omega \right) - \chi _{yyy}\left( {E_y\left( \omega \right)} \right)^2.}$$

11 $$P_z\left( {2\omega } \right) = \chi _{zxx}\left[ {\left( {E_x\left( \omega \right)} \right)^2 + \left( {E_y\left( \omega \right)} \right)^2} \right] + \chi _{zzz}\left( {E_z\left( \omega \right)} \right)^2\chi _{zzz}\left( {E_z\left( \omega \right)} \right)^2 + \chi _{zxx}\left( {E_y\left( \omega \right)} \right)^2.$$

By substituting [6], [7] and [8] into [9], [10] and [11], we make the dependence on α explicit:

12 $$P_x\left( {2\omega } \right) \cong 0.$$

13 $$P_y\left( {2\omega } \right) = \chi _{xxz}E\left( \omega \right)\sin 2\left( \alpha \right) - \chi _{yyy}E\left( \omega \right)\left( {\sin \left( \alpha \right)} \right)^2.$$

14 $$P_z\left( {2\omega } \right) = \chi _{zzz}E\left( \omega \right)\left( {\cos \left( \alpha \right)} \right)^2 + \chi _{zxx}E\left( \omega \right)\left( {\sin \left( \alpha \right)} \right)^2.$$

Then, the resulting SHG signal in the far field reads:

15 $$\begin{array}{l}I^{2\omega } = C\left\{ {\left[ {\left( {\chi _{xxz}E\left( \omega \right)\sin 2\left( \alpha \right) - \chi _{yyy}E\left( \omega \right)\left( {\sin \left( \alpha \right)} \right)} \right.^2} \right]^2 + \left[ {\chi _{zzz}E\left( \omega \right)\left( {\cos \left( \alpha \right)} \right)} \right.^2} \right.\\ \left. {\left. { + \chi _{zxx}E\left( \omega \right)\left( {\sin \left( \alpha \right)} \right)^2} \right]^2} \vphantom{\left[ {\left( {\chi _{xxz}E\left( \omega \right)\sin 2\left( \alpha \right) - \chi _{yyy}E\left( \omega \right)\left( {\sin \left( \alpha \right)} \right)} \right.^2} \right]^2}\right\}.\end{array}$$

By fitting the experimental results to equation [15], we are able to probe different properties of corneal collagen, i.e., whether the resonance frequency of the harmonophores is greater enough than the excitation frequency to meet Kleinman condition and, more importantly, whether the supramolecular assembly of collagen fibrils matches an helicoidal or a staggered side-by-side distribution.

By considering that element χ_yyy_ takes a value that departs from zero as the trigonal symmetry prevails over cylindrical symmetry, our definition of S (S = |d_**22**_|/d_**31**_ **=** |χ_yyy_ |/χ_zxx_, see previous paragraph for details) emphasizes the break of cylindrical symmetry and serves as a convenient metrics to gauge the extent of twisting of collagen lamellae. This parameter is useful for characterizing the supramolecular symmetry of corneal collagen and, in particular, for discriminating sutural lamellae, i.e., a class of collagen lamellae that are localized in the anterior portion of corneal stroma in normal human tissue, run in random directions and often branch out and intertwin in irregular patterns^33^.

### Multimodal Brillouin-Raman imaging setup

We used a custom-made micro-spectroscopic setup able to simultaneously perform Brillouin and Raman imaging. Figure 5e schematically shows the experimental arrangement: the light source is a single mode solid-state laser (Spectra Physics Excelsior, Spectra Physics K.K., Santa Clara, CA, US) emitting a single longitudinal mode at 532 nm. Light was coupled to a confocal microscope (CM- JRS Scientific Instruments) and focused onto the sample by a M-Plan Apo ×20 objective lens (Mitutoyo, Kawasaki, Japan) with a very long working distance (WD = 20 mm) and a numerical aperture of 0.42, yielding a spatial resolution on the sample of ~2 × 2 × 10 μm^3^.

The objective lens was used both to focus and to collect the back-scattered light, which was separated according to its frequency by a tuneable ultrasteep short-pass filter (TEF, SP01-561RU, Semrock Inc., Rochester, NY, US). The anti-Stokes quasi-elastically scattered component was transmitted to a TFP-2 HC Fabry–Perot interferometer (JRS Scientific Instruments, Table Stable Ltd., Mettmenstetten, Switzerland), while Stokes deeply inelastically scattered light was reflected towards a iHR320 Triax Raman Spectrometer (RM-Horiba Ltd., Kyoto, Japan). The TFP-2 HC is the high contrast variant of the original Sandercock type tandem multi-pass interferometer and provides unprecedented contrast greater than 10^15^ with frequency resolution of 100 MHz.

For 2D imaging, the Raman and Brillouin spectra were acquired from each point by translating the sample stepwise using a three-axis piezoelectric translation stage (PI 611-3 S Nanocube XYZ, Physik Instrumente (PI) GmbH & Co. KG, Karlsruhe, Germany) with resolution of 0.01 μm and a motion range of 100 µm per axis. For Brillouin-Raman measurements the acquisition time was 65 s per point and the laser power was about 10 mW on the sample.

### Brillouin-Raman acquisitions and analyses

In order to execute sequences of simultaneous Raman/Brillouin measurements synchronized with sample positioning, a dedicated routine was developed using LabSpec 5 software (Horiba Ltd., Kyoto, Japan) for acquisition of Raman spectra and JRS GHOST 7 (JRS Scientific Instruments, Table Stable Ltd., Mettmenstetten, Switzerland) for Brillouin spectra.

Brillouin spectroscopy deals with light scattered by acoustic phonons thermally activated in the material. In essence, Brillouin spectra consist of peaks symmetrically shifted in frequency with respect to the incident light by an amount, *ω*_b_, that relates to M′, i.e., the real part of the longitudinal elastic modulus, through the equation *ω*_b_ = *q*(M′/*ρ*)^0.5^, *q* being the exchanged momentum. The values of *ω*_b_ can be extracted by fitting the intensity *I*(*ω*) of Brillouin peaks by the damped harmonic oscillator (DHO)^21^ function:

16 $$I\left( \omega \right) = \frac{{I_0}}{\pi }\frac{{\omega _{\mathrm{b}}^2{\mathrm{\Gamma }}_{\mathrm{b}}}}{{(\omega ^2 - \omega _{\mathrm{b}}^2)^2 + \omega ^2{\mathrm{\Gamma }}_{\mathrm{b}}^2}}.$$

where 2Γ_b_ is the peak width.

Raman spectroscopy probes light inelastically scattered by inter and intra-molecular vibrational modes of the chemical species present within the sample. Thanks to its high molecular specificity, Raman spectroscopy is suitable to analyse the chemical composition of materials. Peak intensities are proportional to the concentration of molecular species multiplied by their optical activity. Therefore, probing the modulation of peak intensities in different regions of a sample provides images with contrast related to molecular composition. However, Raman spectra enjoy no internal spectroscopic marker for their normalization. To eliminate possible spurious effects related to (i) fluctuations of the intensity of the laser, (ii) slight differences in the optical absorbance of the sample and (iii) slight modifications in the scattering volume, the use of a normalization peak is a common solution. This approach makes it possible to probe relative concentration changes of the same chemical species in different points of the sample or to follow relative concentration changes among different species. In our case, we chose the amide I signal as normalization peak, which has established as gold standard both for IR^59^ and Raman^7^ studies. The analysis of Raman data was performed by using dedicated Fortran and Matlab codes. In order to accurately estimate the area of the analysed peaks, a background fitted with an sp-line accounting for dark counts and weak luminescence was subtracted from each acquisition.

### TEM imaging

Samples were further imaged by a CM12 Transmission Electron Microscope (Philips, Amsterdam, the Netherlands), operating at 80 kV and equipped with a MegaViewG2 camera (Olympus Corporation, Tokyo, Japan). For TEM imaging, corneal samples were divided into 1 mm^2^ surface area fragments, which were washed twice with phosphate-buffered saline (0.1 M, pH 7.2) and then fixed with fresh glutaraldehyde (2.5%) in the same buffer at 4 °C for 12 h. Then all samples were sequentially washed three times (15 min) with phosphate-buffered saline, post-fixed with osmium tetroxide (1.0%) in the same buffer for 1 h, washed twice with the same buffer (15 min each), dehydrated with 30, 40 and 50% ethanol for 10 min, then 70% ethanol for 20 min, then 80, 95% and pure ethanol for 10 min and with pure propylene oxide for 10 min and finally infiltrated in a Spurr resin^60^. Ultrathin sections of infiltrated samples were collected and deposited onto carbon-coated copper grids with 200 mesh size, stained with uranyl acetate in methanol (50%) for 5 min and with lead citrate for 8 min^61^. Lead citrate was prepared as prescribed by Venable and Goggeshall^62^. All washing procedures were carried out with CO_2_-free water upon bubbling with nitrogen for 30 min.

### Reporting summary

Further information on experimental design is available in the Nature Research Reporting Summary linked to this article.

## Supplementary information

Supplementary Information

Reporting Summary

## Data Availability

Software and algorithms used for analyzing data have been developed using LabVIEW, Fortran and Matlab codes. The routines used are available from the corresponding authors on reasonable request.

## Data availability

The datasets generated during and/or analysed during the current study are available from the corresponding authors on reasonable request.
