# Supplementary material for: Morpho-mechanics of human collagen superstructures revealed by all-optical correlative micro-spectroscopies
Source: Commun Biol. 2019 Mar 26;2:117. doi: 10.1038/s42003-019-0357-y (PMC6435656; doi:10.1038/s42003-019-0357-y)
Supplement: Supplementary file 1 — Supplementary Information [file 42003_2019_357_MOESM1_ESM.pdf]

Supplementary Information

Supplementary Figures

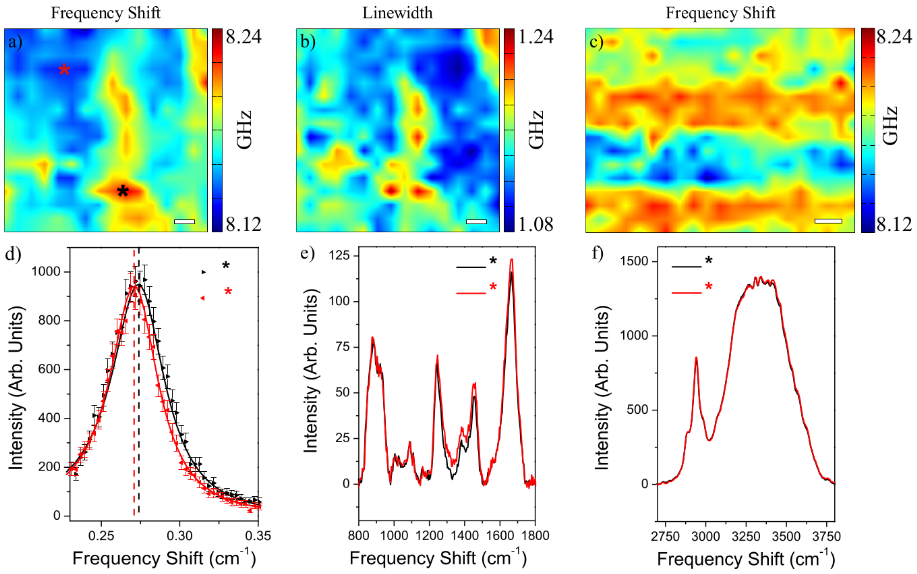

Supplementary Figure 1. Brillouin micro-spectroscopy on additional corneal samples. a) Brillouin map obtained on a fixed corneal sample. b) Brillouin line width relative to panel a c) Brillouin map obtained on a non-fixed corneal sample after sectioning. The mechanical modulation is still present.). d), e) and f) Brillouin and Raman spectra from the voxels marked with asterisks of the respective colours in panel a).

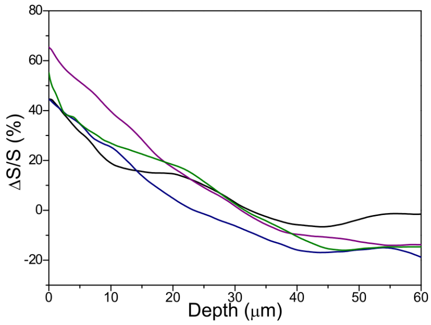

Supplementary Figure 2. P-SHG analysis on additional corneal samples. Behaviour of the symmetry parameter versus stromal depth obtained from the analysis of P-SHG data acquired on four different human corneas.

Supplementary Discussion

Brillouin micro-spectroscopy and SHG measurements on more corneal samples

To challenge the reproducibility of our results, we applied our techniques to different corneal samples. In particular, micro-Brillouin spectroscopy was applied to two more samples exhibiting once again a clear signature of elastic heterogeneities in 2D maps. One of these samples was analysed without using any fixation procedure (Supplementary Figure 1a), thus clearly demonstrating that the revealed mechanical modulation is a genuine feature associated to the collagen structures and not an artefact depending on sample preparation.

The P-SHG analysis was performed on four more human corneas. All samples exhibit a similar variation of the symmetry parameter versus stromal depth. The distinctive decreasing behaviour of ΔS/S is reported in Supplementary Figure 2.
