# Supplementary material for: Morpho-mechanics of human collagen superstructures revealed by all-optical correlative micro-spectroscopies
Source: Commun Biol. 2019 Mar 26;2:117. doi: 10.1038/s42003-019-0357-y (PMC6435656; doi:10.1038/s42003-019-0357-y)
Supplement: Supplementary file 2 — Reporting Summary [file 42003_2019_357_MOESM2_ESM.pdf]

nature research

Corresponding author(s): Riccardo Cicchi, Silvia Caponi

Reporting Summary

Nature Research wishes to improve the reproducibility of the work that we publish. This form provides structure for consistency and transparency in reporting. For further information on Nature Research policies, see Authors & Referees and the Editorial Policy Checklist.

Statistical parameters

When statistical analyses are reported, confirm that the following items are present in the relevant location (e.g. figure legend, table legend, main text, or Methods section).

n/a	Confirmed	
☐	☒	The exact sample size (n) for each experimental group/condition, given as a discrete number and unit of measurement
☐	☒	An indication of whether measurements were taken from distinct samples or whether the same sample was measured repeatedly
☒	☐	The statistical test(s) used AND whether they are one- or two-sided<br>*Only common tests should be described solely by name; describe more complex techniques in the Methods section.*
☒	☐	A description of all covariates tested
☒	☐	A description of any assumptions or corrections, such as tests of normality and adjustment for multiple comparisons
☐	☒	A full description of the statistics including central tendency (e.g. means) or other basic estimates (e.g. regression coefficient) AND variation (e.g. standard deviation) or associated estimates of uncertainty (e.g. confidence intervals)
☒	☐	For null hypothesis testing, the test statistic (e.g. F, t, r) with confidence intervals, effect sizes, degrees of freedom and P value noted<br>*Give P values as exact values whenever suitable.*
☒	☐	For Bayesian analysis, information on the choice of priors and Markov chain Monte Carlo settings
☒	☐	For hierarchical and complex designs, identification of the appropriate level for tests and full reporting of outcomes
☒	☐	Estimates of effect sizes (e.g. Cohen's d, Pearson's r), indicating how they were calculated
☐	☒	Clearly defined error bars<br>*State explicitly what error bars represent (e.g. SD, SE, CI)*

Our web collection on statistics for biologists may be useful.

Software and code

Policy information about availability of computer code

Data collection	In order to execute sequences of simultaneous Raman/Brillouin measurements synchronized with sample positioning, a software scripting system was developed using LabSpec 5 software (Horiba Ltd., Kyoto, Japan) for acquisition of Raman spectra and JRS GHOST 7 (JRS Scientific Instruments, Table Stable Ltd., Mett-menstetten, Switzerland) for Brillouin spectra. Custom LabView routine was used for controlling the custom-made SHG microscope and for acquiring P-SHG data.
Data analysis	Home made fortran code is used for the Brillouin and Raman data analysis. Custom LabView routine was used for analysing P-SHG data. Gnuplot or Origin are used to plot the data.

For manuscripts utilizing custom algorithms or software that are central to the research but not yet described in published literature, software must be made available to editors/reviewers upon request. We strongly encourage code deposition in a community repository (e.g. GitHub). See the Nature Research guidelines for submitting code & software for further information.

Data

Policy information about availability of data

All manuscripts must include a data availability statement. This statement should provide the following information, where applicable:

- Accession codes, unique identifiers, or web links for publicly available datasets
- A list of figures that have associated raw data
- A description of any restrictions on data availability

The datasets generated during and/or analysed during the current study are available from the corresponding authors on reasonable request.

Field-specific reporting

Please select the best fit for your research. If you are not sure, read the appropriate sections before making your selection.

☒ Life sciences ☐ Behavioural & social sciences ☐ Ecological, evolutionary & environmental sciences

For a reference copy of the document with all sections, see nature.com/authors/policies/ReportingSummary-flat.pdf

Life sciences study design

All studies must disclose on these points even when the disclosure is negative.

Sample size	We report a proof-of-principle experiment in which the ability of the proposed innovative method is tested investigating an ex-vivo coneal human tissue probing diffent parts and different depths in the central part of the cornea. A total of 4 corneal samples were examined.
Data exclusions	we did not exclude any data.
Replication	The joint Brillouin and Raman analysis is repeated at different depths and correlated with the SHG analysis acquired on the same position.<br>The Brillouin and Raman analysis was performed investigating 4 regions of about 100µmx100µm sampling the cornea with 33 x 33 (step 3 µm) or 25 x 25 (step 4µm) points.<br>In each point, the Raman and Brillouin spectra are acquired always more than 30 s, to obtain good signal to noise ratio and an excellent fit quality.<br>A total of 4 corneal samples were examined.
Randomization	Randomization does not apply to this study, considering that the samples were not allocated into experimental groups.
Blinding	The investigators were not blinded to group allocation.

Reporting for specific materials, systems and methods

Materials & experimental systems

n/a	Involved in the study
☐	☒ Unique biological materials
☒	☐ Antibodies
☒	☐ Eukaryotic cell lines
☒	☐ Palaeontology
☒	☐ Animals and other organisms
☒	☐ Human research participants

Methods

n/a	Involved in the study
☒	☐ ChIP-seq
☒	☐ Flow cytometry
☒	☐ MRI-based neuroimaging

Unique biological materials

Policy information about availability of materials

Obtaining unique materials	Healthy cornea were purchased from Veneto Eye Bank Foundation (Venice, Italy) and selected among those not suitable for transplantation.
